# Effects of *Cordyceps cicadae* Polysaccharide on Gut Microbiota, the Intestinal Mucosal Barrier, and Inflammation in Diabetic Mice

**DOI:** 10.3390/metabo15010008

**Published:** 2025-01-01

**Authors:** Lijia Sun, Huaibo Yuan, Huiqing Ma, Yani Wang

**Affiliations:** School of Food and Biological Engineering, Hefei University of Technology, No. 193, Tunxi Road, Hefei 230009, China; sunlijia0222@163.com (L.S.); 18756981958ma@gmail.com (H.M.); slj19811715271@gmail.com (Y.W.)

**Keywords:** *Cordyceps cicadae* polysaccharides, diabetes mellitus, intestinal flora, intestinal mucosal barrier, inflammation

## Abstract

**Background:** Polysaccharides produced by the edible fungus *Cordyceps cicadae* can regulate blood sugar levels and may represent a suitable candidate for the treatment of diabetes and its complications. However, there is limited information available about the mechanism of how *C. cicadae* polysaccharide (CCP) might improve diabetic conditions. **Methods:** This study investigated its effects on the intestinal microbiota, intestinal mucosal barrier, and inflammation in mice with type 2 diabetes mellitus (T2DM) induced by streptozotocin, and its potential mechanisms. **Results:** Compared with the DC (diabetes model control group), CCPH oral treatment significantly increased the number of beneficial bifidobacteria, bifidobacteria, and lactobacilli (*p* < 0.01), restored the diversity of intestinal microorganisms in diabetic mice, and the proportions of Firmicutes and Bacteroidetes (34.36%/54.65%) were significantly lower than those of the DC (52.15%/32.09%). Moreover, CCPH significantly reduced the content of endotoxin (lipopolysaccharide, LPS) and D-lactic acid(D-LA) (*p* < 0.05), the activities of antioxidant enzymes and total antioxidant capacity were significantly increased (*p* < 0.01), and the content of proinflammatory cytokines TNF-α, IL-6, and IL-1β were reduced by 42.05%, 51.28%, and 52.79%, respectively, compared with the DC. The TLR4/NF-κB signaling pathway, as a therapeutic target for diabetic intestinal diseases, plays a role in regulating the inflammatory response and protecting the intestinal barrier function. Molecular mechanism studies showed that oral treatment with CCPH down-regulated the expression of NF-κB, TLR-4, and TNF-α genes by 18.66%, 21.58%, and 34.87%, respectively, while up-regulating the expression of ZO-1 and occludin genes by 32.70% and 25.11%, respectively. CCPH regulates the expression of short-chain fatty acid levels, increases microbial diversity, and ameliorates mouse colon lesions by inhibiting the TLR4/NF-κB signaling pathway. **Conclusions:** In conclusion, it is demonstrated that in this murine model, the treatment of diabetes with *C. cicadae* polysaccharide can effectively regulate intestinal microbiota imbalance, protect intestinal mucosal barrier function, and reduce inflammation in vivo, suggesting this natural product can provide a suitable strategy for the treatment of T2D-induced gut dysbiosis and intestinal health.

## 1. Introduction

Diabetes is a metabolic disorder characterized by hyperglycemia that results from disturbed insulin secretion or insulin resistance [1]. T2DM in Asian and European populations also manifests with impaired gut microbiota [2,3]. The intestinal microbiota can be considered an endocrine organ, which not only maintains the energy balance in the body but also affects the host’s immune responses. Under the action of environmental factors, the intestinal microbiota may change, which may affect the relationship between bacteria and their host, leading to mild chronic inflammation and disorders associated with obesity [4,5]. In type 2 diabetes mellitus (T2DM), particular microbial variations correlate with insulin resistance, and the composition of the gut microbiota can affect the regulation of blood glucose levels [6]. High levels of glucose and free fatty acids can increase the expression of genes favoring inflammation, exacerbate oxidative stress, and lead to insulin resistance [7]. IL-6 and TNF-a are primary inflammatory factors, and in T2DM, their levels in the intestines are elevated, which can result in pathological damage and diabetic complications [8,9]. One approach to treat T2DM aims to regulate the intestinal microbiota toward a healthier composition [10].

The TLR/NF-kB signaling pathway is activated during the progression of host inflammation and immune responses [11]. Toll-like receptors (TLRs) trigger an innate immune response [12], whereby TLR2 mainly detects the diacylated lipopeptides produced by Gram-positive bacteria and TLR4 identifies the lipopolysaccharides (LPSs) of Gram-negative bacteria [13]. In patients and mice with inflammatory bowel disease, intestinal TLR4 expression is increased [14]. These TLRs detect abnormal levels of gut microbes, and their activation enhances the expression of downstream proteins such as MyD88 and NF-kB [15,16,17] and promotes dendritic cell maturation [18]. The secretion of proinflammatory cytokines such as TNF-a disrupts the homeostasis of the intestinal mucosa and exacerbates the inflammatory response. The inflammatory balance can be restored by inhibiting the expression of TLR4 and NF-kB, gradually alleviating the pathological damage to the colon [19].

*Cordyceps cicadae* is a fungus that parasitizes the larvae of insects, mainly locusts, from the order Lepidoptera [20]. In its natural environment, the fungus appears as long and slender stalks that envelop the remains of an infected larva. When mature, the upper part of the stalk develops a spore sac with spores. *C. cicadae* is widely distributed and easily accessible and is safe for human consumption, as demonstrated by historical experience and pharmacological studies [21]. The fungi have multiple beneficial properties, including anti-oxidation [22], anti-inflammatory [23], and anti-tumor [24] activity and the ability to protect the kidney [25] and assist in the regulation of blood sugar levels [26]. The polysaccharides produced by certain fungi can improve intestinal microbial imbalance, and this may point toward potential in the treatment and management of obesity and diabetes. For instance, murine experiments have shown that the polysaccharides derived from Ganoderma lucidum can reduce body weight, inflammation, and insulin resistance, and they reversed gut dysbiosis in high-fat diet-induced obese mice [27]. Similar activities were described for polysaccharides derived from Ophiocordyceps sinensis (also known as Hirsutella sinensis) [28]. In vivo studies have demonstrated that *C. cicadae* polysaccharides can improve T2DM by reducing insulin resistance. This effect may be associated with the activation of the PI3K/Akt pathway [26]. In this study, we extracted and purified *C. cicadae* polysaccharides from the fungus, examined their effects on glycolipid metabolism in T2DM mice, and further investigated the molecular mechanisms of *C. cicadae* polysaccharides in relation to improvements in intestinal flora, the intestinal mucosal barrier, and inflammatory responses.

## 2. Materials and Methods

### 2.1. Preparation of C. cicadae Polysaccharides

*Cordyceps cicadae* fungus in powder form was provided by Anhui Cordyceps Source Biotechnology Co., Ltd. The crude *C. cicadae* polysaccharides were extracted by hot water extraction and ethanol precipitation. *C. cicadae* fungus powder (30 g) was dissolved in 900 mL of distilled water and extracted at 70 °C for 2 h. After filtration, it was transferred to a rotary evaporator (Yalong re-5299, Shanghai, China) and concentrated to 100 mL at 55 °C. Next, 80% (*v*/*v*) ethanol was added to the concentrate at a ratio of 1:4 (*v*/*v*), and the concentrate was incubated overnight at 4 °C and then centrifuged (5000 r/min, 20 min). The precipitate was collected. The precipitate was dissolved in distilled water, activated carbon was added at a ratio of 1%, and the solution was decolorized in a water bath at 60 °C for 2 h. Deproteinization was carried out using the Sevag method [29] (chloroform: n-butanol = 4:1 (*v*/*v*) mixed and shaken well) until the organic layer became transparent. After that, the polysaccharide solution was packed into a dialysis bag (MW: 3500) at 4 °C and dialyzed in tap water and ultrapure water sequentially for 48 h. Finally, the crude polysaccharide powder was obtained by vacuum freeze drying.

The crude polysaccharide powder was prepared into a solution of 10 mg/mL using ultrapure water, and the supernatant was centrifuged at 4 °C (3500 r/min, 10 min). Then, the sample solution was eluted on a DEAE-cellulose 52 column at a flow rate of 1 mL/min to obtain the sample solution, which was dialyzed in tap water and ultrapure water with a dialysis bag (MW: 3500) for 48 h and then concentrated and freeze-dried again to make *C. cicadae* polysaccharide powder.

### 2.2. Induction of Type 2 Diabetes Mellitus in Mice and the Treatment Schedule

1. Sample size and oral administration dose selection

Based on the literature and accumulated laboratory experience, the sample size in the results consistently used by others in this line of research was borrowed, i.e., 6 treatment groups of 10 mice each.

Based on previously reported doses, and after preliminary testing in mice to determine that there were no signs of toxicity (weight loss, dysphoria, decreased activity, or other symptomatic changes), orally administered doses of 100, 200, and 400 mg (kg bw)^−1^ of CCP were selected for further experimentation in the type 2 diabetic mice.

Metformin is a classic hypoglycemic agent that has been in clinical use for more than 60 years. Mice treated with metformin as a standard drug by oral gavage as a positive control group were used to assess the efficacy of CCP’s effects on the gut microbiota, the intestinal mucosal barrier, and inflammation in type 2 diabetic mice.

2. Grouping and treatment

All animal experiments received approval from the Biomedical Ethics Committee of Hefei University of Technology. Sixty specific pathogen-free (SPF) male mice (body weight: 20 ± 2 g) were commercially purchased and kept in the animal facility at 22 °C with alternating 12 h day and night cycles. Following a week of adaptation with normal feed, the mice were randomly divided into 6 groups of 10 animals. The normal control (NC) group was fed a regular diet, while the other five groups received a high-fat diet for two weeks. Then, after fasting for 12 h, these animals were injected intraperitoneally with 100 mg (kg bw)^−1^ of streptozotocin to induce TDM. After three days, following a 12 h fasting period, blood was drawn from the tail tip, and blood sugar levels were measured using a glucose meter. Blood sugar levels above 11.1 mmol/L, combined with symptoms of polyuria, obesity, and thinning hair, demonstrated that T2DM had been successfully induced.

All animals were then fed an ordinary diet. Of the five groups of mice in which T2DM was successfully induced, one group served as a diabetes model control group (DC) without any treatment. The other four experimental groups received the following treatment by daily oral gavage for a period of 4 weeks: 200 mg (kg bw)^−1^ of metformin hydrochloride (PC), a low-dose group of 100 mg (kg bw)^−1^ of CCP (CCPL), a medium-dose group of 200 mg (kg bw)^−1^ of CCP (CCPM), and a high-dose group of 400 mg (kg bw)^−1^ of CCP (CCPH).

### 2.3. Intestinal Microbiota Culture

On the last day, after four weeks of treatment, fresh droppings were collected, and one part was rapidly frozen in liquid nitrogen and stored at −80 °C for later analysis. Another part was cultured for bacterial enumeration. For this, 0.1 g of feces was suspended in 0.9 mL of sterile water, 10-fold serially diluted, and plated on suitable selected media for the growth of Bifidobacterium, Lactobacillus, and Bacteroides [30]. Plates were incubated at 36 °C for 2–3 days, and colony counts were used to calculate the number of colony-forming units (CFU) per gram of feces.

### 2.4. Determination of Biochemical Indicators

After the experiment, the mice were anesthetized with ether and euthanized by cervical spine dislocation. Blood was collected from the eyeballs, transferred to 1.5 mL sterile centrifuge tubes, and left at room temperature for 30 min. After centrifugation (15 min at 3000 rpm and 4 °C), the serum supernatant was collected and stored at −20 °C. The colon was extracted from each animal, and after the removal of feces (which was collected, frozen in liquid nitrogen, and stored at −80 °C for bacterial analysis), the colon was washed with phosphate-buffered saline (PBS, 0.01 M, pH 7.4). A small sample (0.1 g) of colon tissue was homogenized in 0.9 mL of PBS and centrifuged (10 min at 5.000 g and 4 °C), after which the supernatant was stored at −80 °C.

The amounts of endotoxin (LPS), D-lactate (D-LA), tumor necrosis factor (TNF-a), interleukin-6 (IL-6), and interleukin-1b (IL-1β) in the serum as well as the enzyme activity of superoxide dismutase (SOD), catalase (CAT), and total antioxidant capacity (T-AOC) in the homogenized colon tissue supernatant were measured using ELISA kits (China Co., Ltd., Quanzhou, China).

### 2.5. Determination of Short-Chain Fatty Acids (SCFAs) in Feces

SCFAs were extracted from the stored feces; fecal samples were thawed at 4 °C and added to 0.4 mL of 50% (*v*/*v*) acetonitrile–water and vortexed for 1 min. Ultrasound treatment was performed at 4 °C for 30 min. After this, 0.4 mL of a solution containing 200 mM 2-nitrophenylhydrazine (3-NPH), 120 mM EDC, and 6% pyridine (2:1:1 *v*/*v*/*v*) was added, vortexed for 1 min, and incubated at 40 °C for 1 h, with intermittent shaking every 5 min. Following centrifugation for 15 min at 12,000 rpm and 4 °C, the supernatant was filtered through a 0.22 mm filter before GC-MS/MS analysis.

A gas chromatograph (Trace 1310) was used that was coupled to a mass spectrometer (TSQ 8000 Evo, Thermo Scientific Technologies, Austin, TX, USA) and fitted with an Agilent HP-Innowax GC column (30 m 0.25 mm ID 0.25 mm). An injection volume of 1 mL (injection port: 240 °C) in splitless injection mode was used, and helium was the carrier gas. The temperature settings during separation were as follows: 50 °C for 1 min, then an increase to 180 °C at a rate of 10 °C/min, followed by an increase to 240 °C at a rate of 40 °C/min, and finally, the temperature was held constant at 240 °C for 3 min. Mass spectra were acquired with an electron ionization voltage of 70 eV [31].

### 2.6. Illumina Sequencing of the 16SrRNA Gene

DNA was extracted from the stored murine feces using an OMEGA reagent kit. The integrity of the isolated DNA was checked by agarose gel electrophoresis. The V3-V4 region of the 16S rRNA gene was amplified by PCR using primers 341F (5′-CCTACGGGNGGCGCAG-3′) and 805R (5′-GACTACHVGGGTATCTAATCC-3′). Following two rounds of PCR amplification, the PCR products were checked by agarose electrophoresis, excised from the gel, and purified. High-throughput sequencing and analysis were conducted as described elsewhere [32].

The reads were attributed to operational taxonomic units (OTUs) and species, and the alpha and beta diversity indices were calculated. A pie chart was constructed to illustrate the relative abundance of different taxa. A PCA map was used to analyze the similarity in microbial composition in different groups.

### 2.7. Intestinal Pathological Examination

The colon tissue of the mice was fixed in 4% paraformaldehyde for 24 h, and after embedding in paraffin, 4 mm-thick sections were cut. These were stained with hematoxylin and eosin (HE) and microscopically examined. Goblet cells were visualized by AB-PAS staining. The micrographs were analyzed with CaseViewer software 2.4.0 (Seville, Hefei, China).

### 2.8. RT-PCR

Colon tissue was homogenized in a sterilized mortar using liquid nitrogen and extracted with TRIzol reagent. The RNA was stored at −80 °C. Copy DNA (cDNA) was produced with a Fsq-301 cDNA reverse-transcribed transcription kit (Shanghai Biotechnology Co., Ltd., Beijing, China). RT-PCR was carried out using Taq PCR Master Mix (Shanghai Biotechnology Co., Ltd., Beijing, China), as listed in Table 1.

### 2.9. Protein Expression

The NF-kB, TLR-4, TNF-a, ZO-1, and mycin concentrations in the mouse colon tissues were determined by ELISA assay (Ruixin Biological Co., Ltd., Hefei, China). All the ELISA kits were 48-well-sized.

### 2.10. Statistical Analysis

Three parallel experiments were conducted for each trial, and the results are expressed as mean ± standard deviation. One-way ANOVA was performed using the IBM SPSS statistics v.26 method, and multiple comparisons between groups were performed using the Duncan method. OriginPro 9.1 software (2021) was used to plot the data, and significance levels of *p* < 0.01 and *p* < 0.05 between groups were reported.

## 3. Results

### 3.1. Cultural Assessment of the Microbial Abundance of Beneficial Bacteria

**Figure 1:**
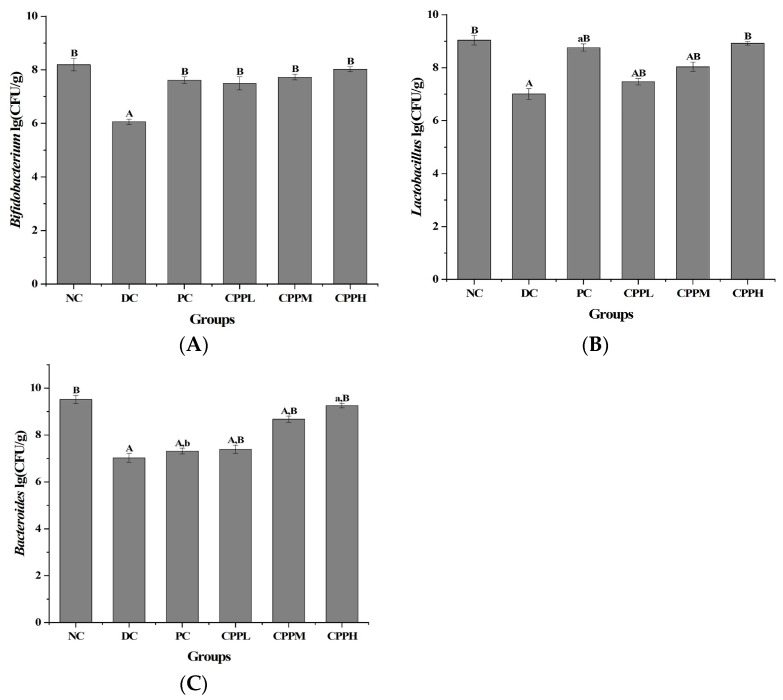
Quantification by cultivation of beneficial bacteria in the intestines of diabetic mice under various treatments (n = 10 per group), with Bifidobacterium (**A**), Lactobacillus (**B**), and Bacteroides (**C**). Data are presented as mean ± standard deviation (SD) values. Statistical significance is indicated as A (*p* < 0.01) and a (*p* < 0.05) vs. the normal control and as B (*p* < 0.01) and b (*p* < 0.05) vs. the diabetic control.

Bifidobacterium, Lactobacillus, and Bacteroides are three beneficial core genera in the intestine, which play an important role in constructing a stable intestinal flora ecology. Among them, Bifidobacterium helps to promote the balance of intestinal flora, inhibit the growth of harmful bacteria, and maintain the micro-ecological balance of the intestinal tract, thus promoting the repair of intestinal mucosal damage. Lactobacillus regulates immune function and maintains intestinal health by lowering serum cholesterol levels and promoting the fermentation of saccharides to produce lactic acid. Bacteroides promote the decomposition of polysaccharides to produce short-chain fatty acids, which promote the body’s glucose metabolism reaction and maintain intestinal homeostasis. The effects of CCP on the intestinal flora ecology of type 2 diabetic mice were evaluated by measuring the abundance of three beneficial bacteria in the feces of mice in different treatment groups. The number of Bifidobacterium, Lactobacillus, and Bacteroides were determined in the fecal droppings of the mice (Figure 1). In comparison to the NC group, the number of these beneficial bacteria was significantly decreased (*p* < 0.01), suggesting induced-T2DM mice suffered from microbial dysbiosis. In the PC group (that had received metformin), the number of Bifidobacterium and Lactobacillus was significantly higher (*p* < 0.01) than in the DC group. Treatment with CCP also elevated the numbers of beneficial Bifidobacterium, Lactobacillus, and Bacteroides (*p* < 0.01) compared to the DC group, and this increase was dose-dependent, suggesting that the treatment could effectively improve the intestinal dysbiosis in the diabetic mice.

### 3.2. Levels of Endotoxin and D-Lactate in the Serum of the Mice

**Figure 2:**
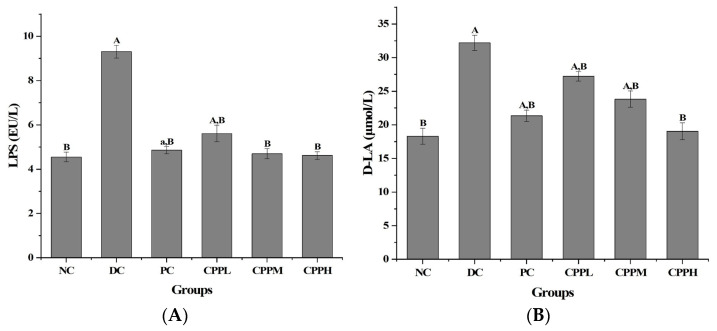
Intestinal mucosal barrier indicators of the diabetic mice (n = 10 per group). (**A**) LPS serum levels and (**B**) lactic acid content. The compared statistical significance is given as a: *p* < 0.05, A: *p* < 0.01; compared with the DC group as B: *p* < 0.01.

The levels of LPS and lactic acid in the serum of the mice were determined post- mortem. LPS is normally present at a low level, but increased concentrations are indicative of a damaged intestinal mucosal barrier and can result in inflammation in the body. As shown in Figure 2A, the amount of serum LPS was significantly higher in the DC group than in the NC group (*p* < 0.01), whereas it was significantly lower in the CCPL, CCPM, and CCPH groups than in the DC group. This indicates that the administration of the fungal polysaccharide had effectively ameliorated the intestinal environment. The LPS levels in the PC group were slightly higher than those in the CCPM and CCPH groups, again illustrating a dose-dependent effect, with the highest dose (CCPH) resulting in similar LPS levels, as observed in the NC group. Thus, the effect was dose-dependent, and the optimal effect was obtained with the highest administered dose of *C. cicadae* polysaccharide, which regulated the return of LPS levels in serum to normal levels.

When the intestinal mucosal barrier is damaged and becomes permeable, lactic acid may enter the blood. To assess this, we also detected and compared changes in serum lactic acid levels (Figure 2B). As expected, the level of lactic acid in the DC group was significantly higher than that in the NC group (*p* < 0.01). Treatment by metformin (in the PC group) or by CCP (at all three tested doses) significantly lowered the lactic acid levels again (*p* < 0.01 compared to DC). A gradual decrease in lactic acid levels was observed in CCPL, CCPM, and CCPH, again illustrating a dose-dependent effect, and the therapeutic effect of CCPH even surpassed that of the positive control group (PC).

### 3.3. Colon Antioxidant Capacity Indicators

**Figure 3:**
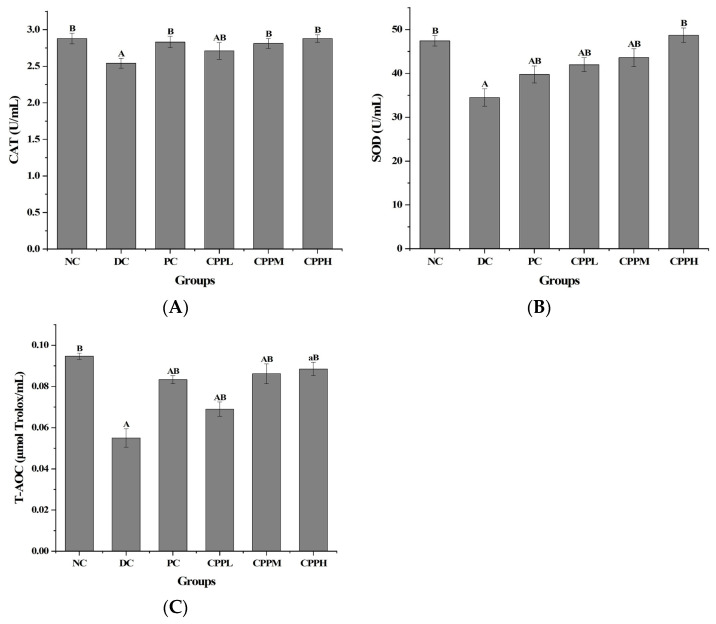
Levels of intestinal antioxidant capacity (*n* = 10 per group). (**A**) CAT level, (**B**) SOD level, (**C**) T-AOC level. Compared with the NC group, a: *p* < 0.05, A: *p* < 0.01; compared with the DC group, B: *p* < 0.01.

The antioxidant levels in the colon tissue of the mice were next analyzed. SOD is an important antioxidant enzyme in organisms, and its role in diabetes is to maintain intracellular oxidative homeostasis and protect cells from inflammation and oxidative stress, which can attenuate oxidative stress-induced cellular damage. CAT is another antioxidant enzyme, mainly present in peroxisomes, where it neutralizes hydrogen peroxide [33]. Inducing T2DM in the mice lowered the antioxidant levels in their colon, as demonstrated in the DC group (*p* < 0.01 compared to the NC group) (Figure 3), while treatment with CCPL, CCPM, and CCPH resulted in higher levels of these antioxidants than in the DC group. The antioxidant capacity of the CCP-treated groups was significantly higher (*p* < 0.01) than that of the DC group and continued to increase with the increasing CCP dose, with the CCPH group being closer to the normal group. These results indicated that treatment with CCP could effectively improve the antioxidant capacity of the murine colon, which probably contributed to reducing intestinal damage in these diabetic mice.

### 3.4. Quantification of Proinflammatory Cytokines

**Figure 4:**
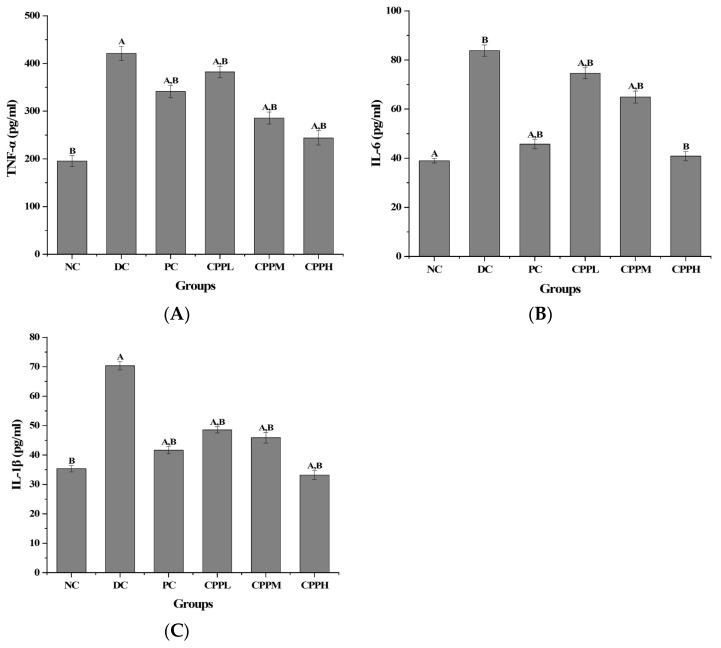
Content of proinflammatory cytokines (**A**–**C**) in diabetic mice (n = 10 per group). Compared with the NC group, A: *p* < 0.01; compared with the DC group, B: *p* < 0.01.

Figure 4 shows the amounts of the proinflammatory cytokines TNF-a, IL-6, and IL-1β in the serum of the animals. Compared to the NC group, the content of these proinflammatory cytokines exhibited a significant increase in the DC group. Their levels were significantly decreased (*p* < 0.01 compared to DC) in the PC, CCPL, CCPM, and CCPH groups. Among them, the CCPH-treated group resulted in the lowest amounts, and a dose-dependent effect was again observed. No significant difference was observed for IL-6 between the CCPH group and the NC group (*p* > 0.05), indicating these levels had been restored to normal. The TNF-a levels in the CCPM and CCPH groups and the concentrations of IL-6 and IL-1β in the CCPH group were all lower than those in the PC group. These findings indicate that *C. cicadae* polysaccharides can effectively mitigate inflammation in diabetic mice.

### 3.5. Short-Chain Fatty Acid (SCFA) Levels

SCFAs play a vital role in supplying energy to intestinal cells, particularly the epithelial cells in the colon. This energy provision is essential for maintaining the integrity and functionality of the intestinal mucosal barrier. The synthesis of SCFAs is pivotal for the development of obesity, diabetes, and various metabolic diseases, and SCFAs are mainly produced by gut microbes during the fermentation of polysaccharides [34]. As illustrated in Table 2, the fecal content of SCFAs in the diabetic mice of the DC group had decreased compared to the NC group (*p* < 0.01); these included acetic, propionic, butyric, isobutyric, valeric, and isovaleric acid. In the PC group, the levels of acetic, butyric, valeric, and isovaleric acid had been restored to those of the NC group; however, propionic acid and isobutyric acid levels were still not normal (*p* < 0.05 compared to NC). In the CCPH group, the levels of propionic, butyric, isobutyric, and valeric acid showed a significant increase compared to the DC group (*p* < 0.01). Of these, the levels of butyric and isobutyric acid were now similar to those of the NC group, although valeric acid and isovaleric acid levels were still different compared to the NC group (*p* < 0.05). Thus, *C. cicadae* polysaccharides were effective in increasing short-chain fatty acid levels in type 2 diabetic mice, which in turn, contributed to the improvement of intestinal health.

### 3.6. Distribution of Gut Microbiota Features in Mice

**Figure 5:**
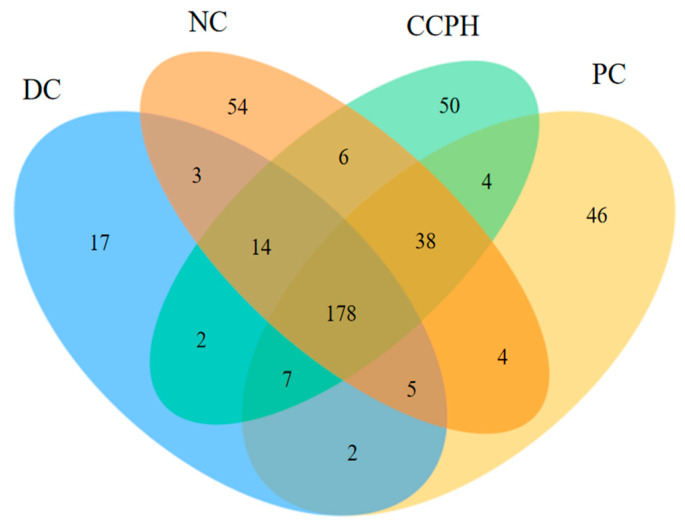
Venn diagram of OTU distribution.

The microbial community present in the colon of the CCPH group was analyzed by amplicon sequencing of the 16S rRNA gene, and this was compared to the findings of the NC, DC, and PC groups. As shown in Figure 5, the number of OTUs shared by the four groups was 178, with similar numbers of OTUs unique to the NC group and the CCPH group. The highest number of OTUs was detected in the NC group, whereas the DC group had the lowest number, indicating that the number of microbial species increased after treatment of the diabetic mice.

### 3.7. Microbial Species Composition Analysis

**Figure 6:**
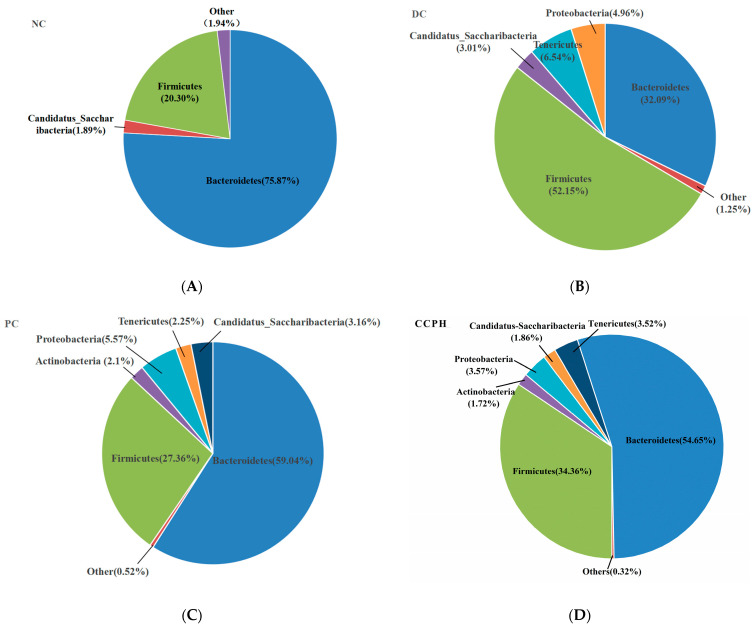
Distribution of microbial communities at the phylum level. (**A**–**D**) microbial species composition from the NC group, DC group, PC group and CCPH group.

Microorganisms play an important role in promoting the metabolism of non-digestible polysaccharides and producing essential vitamins. This microbial activity is instrumental in influencing the development and differentiation of the intestinal epithelium [35]. In particular, the Firmicutes/Bacteroidetes ratio is frequently associated with susceptibility to disease, and this ratio tends to decrease after weight loss in obese patients [36]. The obtained sequence reads were attributed to bacterial phyla to assess this ratio. As shown in Figure 6, the fraction of Firmicutes was 20.30% in the NC group, but it was more than 2.5 times higher (52.15%) in the DC group, close to normal (27.36%) in the PC group, and 34.36% in the CCPH group. Bacteroidetes was present at a fraction of 32.09% in the DC group, 59.04% in the PC group, and 54.65% in the CCPH group. This produced a Firmicutes/Bacteroidetes ratio of 0.27 in the NC group, 1.62 in the DC group, 0.46 in the PC group, and 0.63 in the CCPH group. Thus, the treatment with CCP greatly improved the diabetes-induced intestinal microbial disorder.

### 3.8. Alpha Diversity Analysis

Alpha diversity is typically analyzed using the Shannon, Simpson, and Chao indices, whereby the Shannon index and Simpson index serve as indicators of microbial diversity. A higher Shannon index signifies higher community diversity, whereas a higher Simpson index indicates lower community diversity [37]. The larger the Chao1 index is, the higher the community diversity. It has been shown that the Shannon diversity index and the estimated Chao1 richness were significantly reduced in mice with colitis induced by dextran sulfate sodium (DSS), and Kae effectively prevented the decrease in bacterial community diversity and richness caused by DSS [38]. As shown in Table 3, the DC group exhibits the lowest Chao1 index, indicative of low species richness, the lowest Shannon index, and the highest Simpson index, which in combination, points to the lowest community diversity. Conversely, the NC and CCPH groups have a higher Shannon index and a Simpson index close to 0, suggesting abundant species in the samples [39]. The microbial diversity of the PC group was lower than that of the CCPH group. In summary, CCPH treatment can effectively improve intestinal microbial diversity and reduce intestinal mucosal barrier damage.

### 3.9. Beta Diversity Analysis

**Figure 7:**
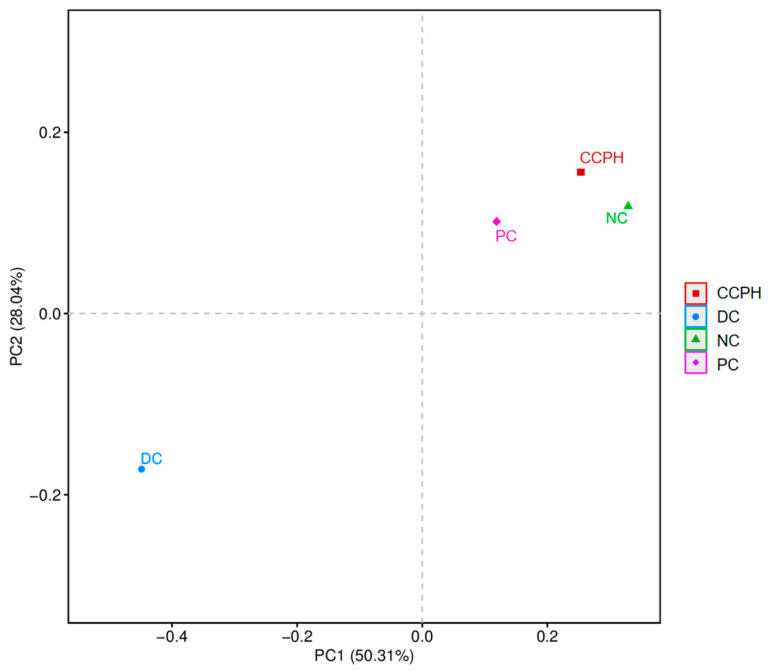
PCA analysis.

Principle component analysis was performed on the sequence data (Figure 7), revealing that the PC group and CCPH group closely resembled the NC group. The proximity of their positions in the plot indicated a higher similarity in microbial composition and a smaller difference between them. Notably, the microbial structure of the DC group differed from that of other groups. This further strengthens the conclusion that the microbial composition in diabetic mice resembled that of the normal group after treatment and suggests that CCP restored the community structure of the intestinal microbiota, alleviating diabetic symptoms.

### 3.10. Pathological Changes in the Colon

**Figure 8:**
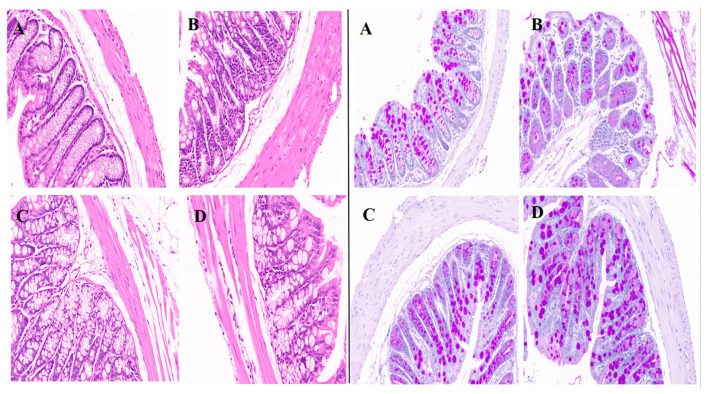
HE (**left**) and AB-PAS (**right**) stained mouse colon sections. (**A**–**D**) Colon from the NC group, DC group, PC group, and CCPH group, respectively.

The colon was microscopically examined to identify any pathological changes in intestinal tissue (Figure 8). HE staining showed that the colon of animals from the NC group was normal, with a clear boundary mucosal edema or the absence of inflammatory cell infiltration with the shedding of local epithelium, and the epithelial cells were arranged in a disorderly manner. The intestinal mucosal structure of the PC group was effectively improved compared with that of the DC group, and the epithelial structure remained relatively intact. In the CCPH group, the mucosal structure was also improved, and only a small number of inflammatory cells had infiltrated. Goblet cells were observed after AB-PAS staining. Compared with the other groups, the inflammatory cells in tissue from the DC group were infiltrated in large quantities and the number of goblet cells was reduced. The number of goblet cells in the PC group was close to that in the NC group, while the highest number of goblet cells was observed in the CCPH group. In summary, after treatment, the pathological features of the colon were significantly reduced, especially in the CCPH group. This clearly indicates that CCPH therapy has a colon protective effect.

### 3.11. Colon mRNA and Protein Expression Levels

**Figure 9:**
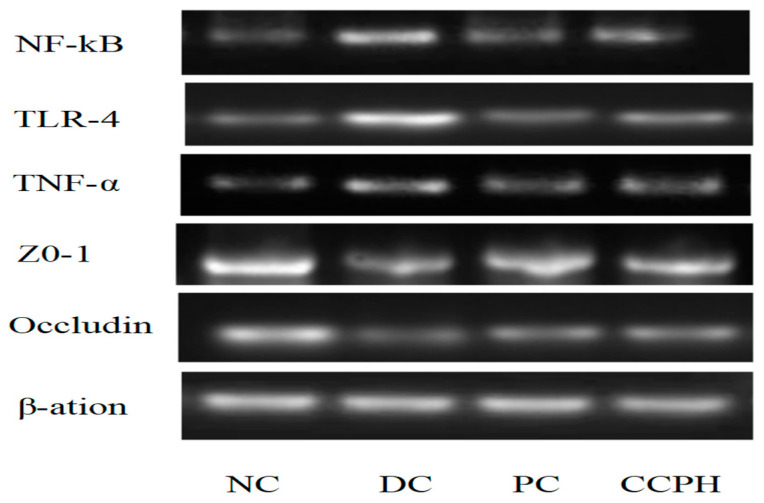
mRNA expression levels of NF-kB, TLR-4, TNF-α, ZO-1, and occludin in the colon.

The transcription levels of proteins participating in the NF-kB/TLR-4 signaling pathway were measured by RT-PCR. As shown in Figure 9, the mRNA levels of NF-kB, TLR-4, and TNF-a were increased in the DC group compared to the NC group, while the mRNA levels of ZO-1 and occludin were lower. After treatment, these gene expression levels were restored close to normal, as the transcription levels of NF-kB, TLR-4, and TNF-a decreased and ZO-1 and occludin increased compared to the DC group. Their transcription levels after treatment tended to align with those of the NC group. The protein expression of NF-kB, TLR-4, TNF-a, ZO-1, and occludin was also assessed, with the results summarized in Table 4. In the DC group, the expression levels of NF-kB, TLR-4, and TNF-a were significantly increased (*p* < 0.01 compared to NC), but their levels decreased after treatment. The expression levels of TLR-4 and TNF-a in the PC group were comparable to those in the normal group, while NF-kB, TLR-4, and TNF-a in the CCPH group were significantly decreased compared to the DC group (*p* < 0.01), indicating that the protein expression levels approached those of the normal group after treatment. In comparison to the DC group, the expression of ZO-1 and occludin in the treatment groups had increased, with ZO-1 in the CCPH and PC groups nearly reaching normal levels (91% and 95% of the NC group, respectively). The expression of occludin in the PC and CCPH groups was 89% and 83% of that of NC, respectively. From this, we conclude that CCP is likely to exert its beneficial effects and improve intestinal health in T2DM mice through the involvement of the NF-kB/TLR-4 signaling pathway.

## 4. Discussion

Numerous natural products have been considered effective treatments for diabetes; their popularity is based on the view that gut microbes can influence the development of T2DM, and their metabolites play an important role in diabetes. The polysaccharide produced by the medicinal fungus *C. cicadae* is such a natural product. It has already been shown that it can reduce blood sugar levels by regulating the community of intestinal bacteria and their metabolites in a murine model in which diabetes was induced by a high-fat diet combined with STZ [40]. The effects of this polysaccharide were further assessed here, using the same mouse model.

The rise in microbial OTU levels may indicate an augmented diversity within the gut microbial community. Elevated microbial diversity is generally deemed advantageous for gut health, as a diverse microbial community is thought to contribute to upholding the integrity and functionality of the intestinal mucosal barrier. The increase in microbial OTU could imply the participation of a greater number of microbial species in various metabolic pathways. This may encompass microbes that influence blood sugar regulation, insulin sensitivity, and other metabolic processes associated with diabetes. Consequently, an increase in microbial OTU levels might impact the metabolic state of the host. Wang et al. studied the effect of CCP on intestinal microflora and found that the ratio of Firmicutes to Bacteroidetes in the treatment group was significantly reduced, indicating that the product has a health-promoting effect [40]. In this study, CCPH treatment also reduced the Firmicutes/Bacteroidetes ratio, and we show that the microbial diversity significantly increased compared to the DC group. These results suggest that CCPH promotes the restoration of a balanced intestinal microbiota, which may improve insulin sensitivity.

An imbalance in intestinal flora in mice can be overcome by dietary intervention to promote the presence and growth of beneficial bacteria (e.g., Bacteroides, Lactobacillus, and Bifidobacterium) while inhibiting the growth of harmful bacteria, including Clostridium species [37]. Another medicinal natural product, Trichosanthin peel powder, has been shown to regulate intestinal flora imbalance and improve intestinal health. Our study showed that treatment with CCP also increased the number of beneficial bacteria in a dose-dependent manner, and their numbers became similar to those of the normal control group. By promoting the growth of beneficial bacteria, CCP may be effective in overcoming intestinal flora disorders and, ultimately, improving the overall intestinal environment.

A study revealed that following propolis intervention (a natural product of bees), treated diabetic rats exhibited a closer resemblance to the control group and the hypoglycemic effect demonstrated by propolis was associated with the amelioration of intestinal microflora disorders. Our analysis of β diversity demonstrated that the microbial structure of the diabetic mice treated with CCP closely resembled that of the NC group. This may be one of the mechanisms by which CCP induces hypoglycemia. Gut bacterial communities play an important role in multiple metabolic disorders, frequently via the production of various metabolites that interact with receptors present in host cells. These receptors can activate or inhibit signaling pathways, with beneficial or harmful effects on the host. A diverse range of bacterial metabolites are involved in these interactions, ranging from small molecules (such as SCFAs) to complex macromolecules (peptidoglycans, LPS). The abundance of bacterial metabolites is influenced by a given microbial composition and is also influenced by dietary and environmental factors [41,42,43]. In this study, the levels of propionic, butyric, isobutyric, and valeric acid in the intestines of CCP-treated mice were significantly higher than those in the DC group. SCFAs serve as regulators of the host’s metabolism, assist in maintaining an intact intestinal mucosal barrier, and down-regulate inflammation [44]. By regulating short-chain fatty acid levels, CCP can effectively improve intestinal flora metabolic disorders, protect the intestinal mucosal barrier, and reduce inflammation [45].

A further role of SCFAs produced by bacteria in the gut is to assist in maintaining an intact intestinal mucosal barrier. This specific and selective barrier is essential to maintain homeostasis. After treatment, the antioxidant activities of enzymes such as CAT, SOD, and T-AOC in the intestinal tract of mice were significantly increased, suggesting that CCP has a certain regulatory effect on the intestinal mucosal barrier function. Examination of the mouse colons showed that the pathological condition of diabetic mice was greatly improved after CCP gavage treatment.

Increased inflammation has been linked to an increase in obesity-related diseases, such as cardiovascular disease [46] and T2DM [47]. Chronic inflammation promotes insulin resistance and β-cell failure, leading to the development of T2DM [48,49]. The concentrations of inflammatory markers, including TNF-a and IL-1β, were significantly elevated in adolescents with T2DM [50], and reduced insulin sensitivity in such patients has been associated with inflammatory mediators, including TNF-a, IL-1β, IL-6, and IL-8 [51]. Therefore, the effect of CCP treatment on these inflammatory mediators was also assessed. Serum levels of TNF-a, IL-1β, and IL-6 were significantly decreased after treatment with CCPH. This suggests that CCPH can alleviate the inflammatory response associated with diabetes. Inflammatory markers serve as therapeutic indicators for the development of T2DM and its complications, and targeting the regulation of the inflammatory system is considered a suitable therapeutic strategy [52].

We further determined the expression levels of important proinflammatory cytokines at the mRNA level. The significant up-regulation of mRNA expression of TLR4, NF-κB, and TNF-α in the DC group, together with elevated protein levels for TLR4 and NF-κB, suggests that a state of chronic low-grade inflammation in the gut had been induced in the animals, which may have contributed to impaired intestinal mucosal barrier integrity or function, allowing LPS to enter the bloodstream, eventually causing systemic inflammation. The involvement of the TLR4/NF-κB signaling pathway may play a pivotal role in this process, as it has been demonstrated that this pathway plays a crucial role in intestinal inflammation [53]. Treatment with CCP by and large reversed these diabetes-induced changes, presumably by inhibiting the TLR4/NF-κB signaling pathway. This may represent a further molecular mechanism underlying the improvement of T2DM following CCP treatment.

In addition, CCP can also reduce systemic inflammation by up-regulating the expression of ZO-1 and occludin and prevents LPS from entering the blood circulation by improving epithelial integrity. This may be a further mechanism by which CCP protects the intestinal mucosal barrier and inhibits inflammation that would contribute to T2DM. Taken together, the reported findings reveal a potential link between gut inflammation and T2DM and provide new insights into how *Cordyceps cicadae* polysaccharides may improve T2DM conditions. These results provide a strong theoretical basis for the development of new treatment strategies.

## 5. Conclusions

In conclusion, this study confirmed that T2DM induced in mice by a high-fat diet and STZ resulted in abnormalities in intestinal microbiota, disrupted the intestinal mucosal barrier, and increased the production of inflammatory factors. Treatment with *Cordyceps cicadae* polysaccharide restored the intestinal microbiota toward a healthier balance of Firmicutes and Bacteroidetes, improved intestinal mucosal barrier integrity and function, and reduced the inflammatory response, thereby alleviating related symptoms in the mice. Its action is based on inhibition of the TLR4/NF-kB signaling pathway. This shows that that CCP has a potential protective effect on intestinal damage in T2DM and may be considered an effective therapeutic natural product for the regulation of intestinal health in T2DM.

## 6. Study Limitations

The limitation of this study is that the efficacy and toxicity of CCP were only preliminarily evaluated in the T2DM mice, but the safety and efficacy of CCP in humans have not been determined by cellular experiments and clinical trials. In vitro, cellular experiments can be conducted subsequently to study the mechanism of action and effects of CCP at the molecular and cellular levels, which will serve as a basis for further research and evaluation. In addition, we found that CCP affects SCFA levels and the intestinal flora structure only in the results of short-term experiments; however, we did not determine whether long-term treatment affects other metabolites, such as amino acids, proteins, and fatty acids, or whether other potential effects exist.

## Figures and Tables

**Table 1:** PCR primer sequence.

Gene	Primer Sequences	Amplicon Length (bp)
NF-KB	PrimerF: CGACTGGTTCACTGCTCCTAATCC PrimerR: ATGCTGGCTGCTGCTTCACTG	382
TLR4	PrimerF: TGTGTCAGTGGTCAGTGTGATTGTG PrimerR: CTGTAGTGAAGGCAGAGGTGAAAGC	225
TNF-α	PrimerF: CACCACGCTCTTCTGTCTACTGAAC PrimerR: TGACGGCAGAGAGGAGGTTGAC	347
ZO-1	PrimerF: GAAGGCGGATGGTGCTACAAGTG PrimerR: AGGCTCAGAGGACCGTGTAATGG	232
occludin	PrimerF: AGTCCACCTCCTTACAGACCTGATG PrimerR: GCCTCCATAGCCACCTCCGTAG	330
β-actin	PrimerF: TGCTGTCCCTGTATGCCTCTGG PrimerR: ACCGCTCGTTGCCAATAGTGATG	348

**Table 2:** Content of short-chain fatty acids in each group (x ± s, n = 10).

Group	Acetic Acid (ng/mg)	Propionic Acid (ng/g)	Butyric Acid (ng/g)	Isobutyric Acid (ng/g)	Valeric Acid (ng/g)	Isovaleric Acid (ng/g)
NC	71.13 ± 1.05 ^B^	3.808 ± 0.1 ^B^	3.568 ± 0.09 ^B^	8.735 ± 0.54 ^B^	10.61 ± 0.56 ^B^	23.17 ± 1.11 ^B^
DC	62.95 ± 1.39 ^A^	1.855 ± 0.05 ^A^	2.463 ± 0.13 ^A^	7.153 ± 0.39 ^A^	8.76 ± 0.77 ^A^	18.82 ± 1.34 ^A^
PC	72.63 ± 1.34 ^B^	3.635 ± 0.1 ^a,B^	3.7 ± 0.14 ^B^	7.933 ± 0.37 ^a,b^	10.24 ± 0.60 ^B^	24.96 ± 1.11 ^B^
CCPH	77.46 ± 1.47 ^A,B^	3.78 ± 0.12 ^B^	3.57 ± 0.17 ^B^	9.183 ± 0.45 ^B^	9.7 ± 0.31 ^a,b^	20.77 ± 1.65 ^a^

Compared with the NC group, a: *p* < 0.05, A: *p* < 0.01; compared with the DC. group, b: *p* < 0.05, B: *p* < 0.01.

**Table 3:** Microbial diversity index.

Group	Shannon	Simpson	Chao
NC	4.748 ± 0.184 ^B^	0.015 ± 0.003 ^B^	281.78 ± 17.27 ^B^
DC	3.840 ± 0.123 ^A^	0.875 ± 0.004 ^A^	202.15 ± 18.12 ^A^
PC	4.283 ± 0.196 ^AB^	0.025 ± 0.007 ^a,B^	259.51 ± 12.01 ^B^
CCPH	4.81 ± 0.430 ^B^	0.016 ± 0.007 ^B^	278.19 ± 15.68 ^B^

Compared with NC group, a: *p* < 0.05, A: *p* < 0.01; compared with DC group, B: *p* < 0.01.

**Table 4:** Protein expression of mice in each group (x ± s, n = 10).

Group	NF-kB (pg/mL)	TLR-4 (ng/mL)	TNF-α (pg/mL) (pg/mL)	ZO-1 (ng/mL)	Occludin (ng/mL)
NC	137.02 ± 6.89 ^B^	2.683 ± 0.23 ^B^	50.39 ± 4.37 ^B^	35.31 ± 1.64 ^B^	1.342 ± 0.15 ^B^
DC	202.74 ± 3.57 ^A^	4.527 ± 0.27 ^A^	89.47 ± 4.85 ^A^	24.17 ± 2.51 ^A^	0.893 ± 0.16 ^A^
PC	152.84 ± 6.21 ^A,B^	2.822 ± 0.21 ^B^	57.35 ± 4.37 ^a,B^	33.63 ± 1.44 ^B^	1.188 ± 0.12 ^B^
CCPH	164.91 ± 6.35 ^A,B^	3.55 ± 0.22 ^A,B^	58.27 ± 4.33 ^A,B^	32.08 ± 2.72 ^a,B^	1.117 ± 0.16 ^a,b^

Compared with the NC group, A: *p* < 0.01, a: *p* < 0.05. Compared with the DC group, B: *p* < 0.01, b: *p* < 0.05.

## Data Availability

Data are contained within the article.
