# Correction: Sun et al. Effects of *Cordyceps cicadae* Polysaccharide on Gut Microbiota, the Intestinal Mucosal Barrier, and Inflammation in Diabetic Mice. *Metabolites* 2025, *15*, 8

**DOI:** 10.3390/metabo16040233

**Published:** 2026-03-31

**Authors:** Lijia Sun, Huaibo Yuan, Huiqing Ma, Yani Wang

**Affiliations:** School of Food and Biological Engineering, Hefei University of Technology, No. 193, Tunxi Road, Hefei 230009, China; sunlijia0222@163.com (L.S.); 18756981958ma@gmail.com (H.M.); slj19811715271@gmail.com (Y.W.)

The authors would like to make the following correction to their published paper [1]. The change is as follows:

Reference [11] in the original publication is incorrectly cited and needs to be replaced with the following reference:

11. Nedosugova, L.V.; Markina, Y.V.; Bochkareva, L.A.; Kuzina, I.A.; Petunina, N.A.; Yudina, I.Y.; Kirichenko, T.V. Inflammatory Mechanisms of Diabetes and Its Vascular Complications. *Biomedicines* **2022**, *10*, 1168.

The authors state that the scientific conclusions are unaffected. This correction was approved by the Academic Editor. The original publication has also been updated.

## References

[1] Sun L., Yuan H., Ma H., Wang Y. (2025). Effects of Cordyceps cicadae Polysaccharide on Gut Microbiota, the Intestinal Mucosal Barrier, and Inflammation in Diabetic Mice. Metabolites.

